# Estimation of C* Integral for Mismatched Welded Compact Tension Specimen

**DOI:** 10.3390/ma14247491

**Published:** 2021-12-07

**Authors:** Marko Katinić, Dorian Turk, Pejo Konjatić, Dražan Kozak

**Affiliations:** Mechanical Engineering Faculty, University of Slavonski Brod, Trg Ivane Brlic Mazuranic 2, 35000 Slavonski Brod, Croatia; dturk@unisb.hr (D.T.); pkonjatic@unisb.hr (P.K.); dkozak@unisb.hr (D.K.)

**Keywords:** creep, C* integral, mismatched weld, CT specimen, finite element analysis

## Abstract

The C* integral for the compact tension (CT) specimen is calculated using the estimation equation in ASTM E1457-15. This equation was developed based on the assumption of material homogeneity and is not applicable to a welded CT specimen. In this paper, a modified equation for estimating the C* integral for a welded compact tension (CT) specimen under creep conditions is proposed. The proposed equation is defined on the basis of systematically conducted extensive finite element (FE) analyses using the ABAQUS program. A crack in the welded CT specimen is located in the center of the heat-affected zone (HAZ), because the most severe type IV cracks are located in the HAZ. The results obtained by the analysis show that the equation for estimating the C* integral in ASTM E1457-15 can underestimate the value of the C* integral for creep-soft HAZ and overestimate for creep-hard HAZ. Therefore, the proposed modified equation is suitable for describing the creep crack growth (CCG) of welded specimens.

## 1. Introduction

Welding technology is one of the most common ways of joining metals in the chemical and petrochemical industries. However, welds may contain imperfections such as lack of fusion, under-cuts, porosity, and slag inclusion of certain height and length at certain locations in the weld [1,2]. Such irregularities in welds usually act as crack initiation sites. The failure of any of the welded joints in the process industry is uncomfortable at best and can lead to catastrophic accidents at worst. For safe and reliable operation of welded structures, periodic non-destructive testing and appropriate assessment of structural integrity should be performed.

There are increasing demands for reducing CO_2_ emissions in the chemical and petrochemical industries and in thermal power plants. Increasing the operating temperatures of the plant increases the energy efficiency, thus reducing CO_2_ emissions. Higher operating temperatures and higher stresses increase the risk of welded structure failure due to creep conditions. It is therefore necessary to develop a method to predict the CCG in welded joints [3].

It is most likely that failure due to creep of high-temperature components begins in welded joints. The most severe cracks in welded joints are type IV cracks in the HAZ. These cracks occur due to multi-axial stresses caused by the material constraint effect between the welding constituents and large creep strain of the soft HAZ [4]. Many previous studies [5,6,7,8] have shown that material constraint plays an important role in CCG in welded joints. The material constraint is due to the mismatch effect of creep properties in the welded joint components. There are a number of experimental and numerical studies on the influence of the mismatch effect on the fracture mechanics parameter C* and the CCG rate in welded joints [9,10,11,12,13,14].

Many studies have shown that in addition to material constraint, the CCG behavior of welded joints is also influenced by geometric constraint [15,16,17]. Li et al. [18] investigated the effect of the interaction of material constraint and geometry constraint on the CCG rate in welded joints. A FE method based on the ductility exhaustion model was used in this study. A FE method was applied to two specimen types (the compact tension and middle crack tension specimen) with different material mismatches. The research established the existence of the effect of the interaction between material and geometry constraints on the CCG rate. Under the condition of low geometry constraint, the effects of material constraint on the CCG rate become more obvious [18].

Creep crack growth under widespread steady-state creep conditions is characterized by the C* integral. The C* integral for the compact tension (CT) specimen is calculated using the estimation equation in ASTM E1457-15 [19]. However, this equation was developed based on the assumption of material homogeneity. As the above studies have shown, the influence of material and geometry constraints on C* and the CCG rate of welded joints cannot be ignored. Therefore, the C* estimation equation according to ASTM E1457-15 needs to be modified for application to welded CT specimens. Xuan et al. [3] proposed a model of equivalent material and developed a modified equation for the C* integral applying limit load solutions. However, the HAZ was not taken into consideration in this study.

In this paper, extensive elastic-creep two-dimensional finite element analysis was performed for welded CT samples with different crack lengths located in the HAZ. The general purpose finite element program ABAQUS [20] was used to calculate the C* integral. A systematic parametric study of the dependence of the C* integral on the mismatch of material properties, HAZ width and crack length was performed. Based on the performed analyses, a modified equation for estimating the C* integral for welded CT samples is proposed.

## 2. Numerical Analysis

### 2.1. Geometry and Loading

**Figure 1:**
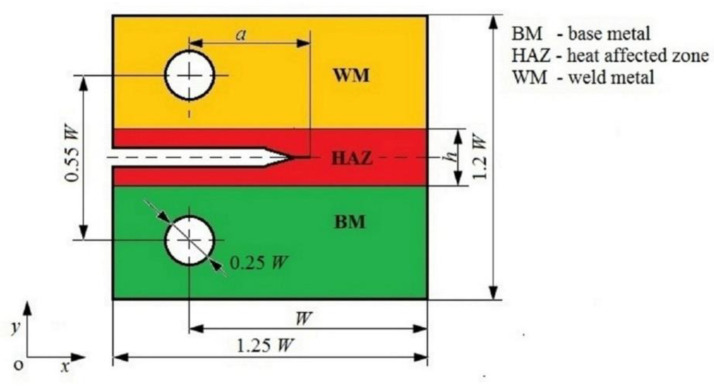
CT specimen of welded joint.

A two-dimensional (2D) high crack-tip constrained plain-strain CT specimen of welded joints was considered. This specimen consists of three materials, as described in Figure 1. The general geometric dimensions of the CT specimen are also shown in Figure 1. The width of specimen was selected to be *W* = 25 mm. The crack is located in the center of HAZ. For the purpose of the investigation, the relative crack length *a/W* was varied from 0.5 to 0.9 with a step of 0.1. Additionally, the relative width of the HAZ *W/h* was varied from 6 to 12 with step 2. All considered specimens were initially loaded with the same stress intensity factor *K* = 460 Nmm^−3/2^.

### 2.2. Material Properties

The elastic-creep material model was used in FE analysis. Generally, the power law relationship is appropriate for modeling the stress–strain response under steady-state creep. Elastic-creep constitutive relation is as follows:

(1) $$\dot{\varepsilon }=\frac{\dot{\sigma }}{E}+A\sigma^{n}\;,$$

where $\dot{\varepsilon }$. is the uniaxial strain rate, $\dot{\sigma }$. is the uniaxial stress rate, and *E* is Young’s modulus. *A* and *n* are the creep constant and exponent, respectively. Young’s modules and Poisson’s ratio for HAZ and WM are assumed to be the same as those of BM. These values are as follows: *E* = 175 GPa and *ν* = 0.3. The power law for the three materials (BM, WM, and HAZ) is as follows:

(2) $${\dot{\varepsilon }}_{\mathrm{BM}}=A_{\mathrm{BM}}\sigma^{n_{\mathrm{BM}}},\;\;{\dot{\varepsilon }}_{\mathrm{WM}}=A_{\mathrm{WM}}\sigma^{n_{\mathrm{WM}}},\;\;{\dot{\varepsilon }}_{\mathrm{HAZ}}=A_{\mathrm{HAZ}}\sigma^{n_{\mathrm{HAZ}}}\;,$$

The selected power law parameters for BM are: *A*_BM_ = 1 × 10^−20^ (MPa)^−*n*^h^−1^ and *n*_BM_ = 7. To investigate the influence of material constraints on the C* integral in welded joints, different configurations of material mismatch in creep strain rate of BM, WM and HAZ were designed. The material creep properties for HAZ and WM were chosen so that the creep strain rate was lower or higher than that of BM. This was achieved by varying the constant *A*. The creep exponent *n* for BM, WM and HAZ was identical (*n*_BM_ = *n*_WM_ = *n*_HAZ_ = 7). The effect of the mismatch in the creep properties for WM and HAZ relative to BM is expressed by the mismatch factor. These mismatch factors are defined as follows [18]:

(3) $${\mathrm{MF}}_{\mathrm{WM}}=\frac{A_{\mathrm{WM}}}{A_{\mathrm{BM}}},\;{\mathrm{MF}}_{\mathrm{HAZ}}=\frac{A_{\mathrm{HAZ}}}{A_{\mathrm{BM}}}$$

The selected values of *MF*_WM_ and *MF*_HAZ_ factors are 10, 1 and 0.1, respectively. Thus, in order to investigate the effect of material constraints on the C* integral, nine possible combinations of material mismatch were considered, as shown in Table 1.

### 2.3. Finite Element Analysis

**Figure 2:**
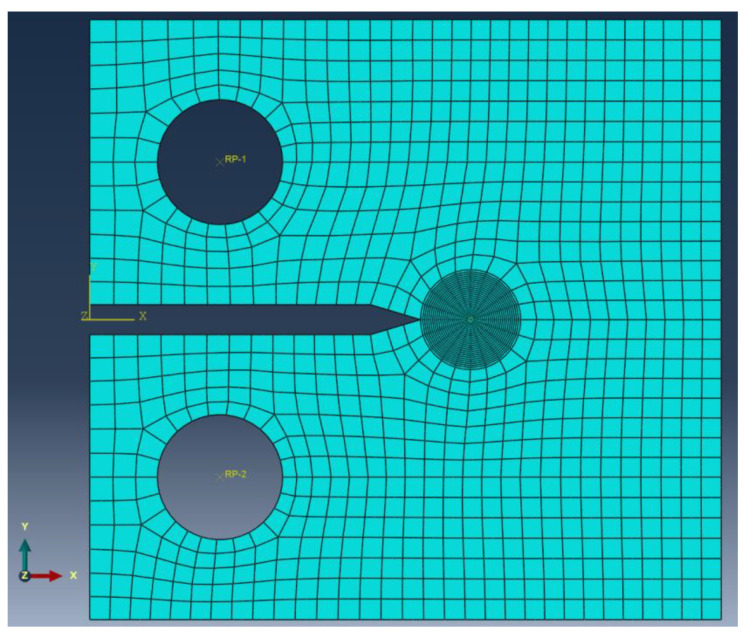
Typical FE mesh for the CT specimen with *a/W* = 0.5.

Extensive elastic-creep FE analyses for welded CT specimens were performed in this study. The general purpose FE program ABAQUS (2016, Dassault Systemes Simulia Corp., Johnston, RI, USA) [20] was used to calculate the C* integral. A seam crack is included in a 2D CT specimen model. The selected crack front is equivalent to the crack tip. The direction of crack propagation at the crack tip is defined using the virtual crack extension direction. A small geometry change continuum FE model was applied. In order to avoid problems associated with incompressibility, 8 node reduced integration elements for 2D plain-strain problems (CPE8R) were used [3]. The elements of innermost ring at the crack tip are degenerated into triangles. The three nodes along one side of the eight-node element are defined so that they share the same geometrical place. Each of the three collapsed nodes can be displaced independently [21]. Figure 2 shows a typical FE mesh for the CT specimen *a/W* = 0.5 and *W/h* = 6. The crack-tip zone is meshed finely. The mesh size at crack-tip region is selected so as to eliminate mesh-sensitivity in determining the stress fields and C* integral. Therefore, the element size selected in all further analyses was 0.05 mm.

The load was applied in the center of the upper hole using the reference point RP-1 and the multi-point constraint (MPC) option within ABAQUS. The direction of the load at the reference point was in the *y* direction. The displacement of the center of the upper hole was constrained in *x* direction [18]. Additionally, the displacement of the center of the lower hole was constrained in *x* and *y* directions [18]. Initially, the model was stress free. The above load was firstly applied instantaneously to the FE model using an elastic calculation at time *t* = 0 [3]. Then, the load was kept constant and a subsequent time-dependent creep calculation was performed. In order to achieve better numerical efficiency for time-dependent creep calculation, a combined implicit and explicit method within ABAQUS was selected. Keeping the load constant, subsequent creep deformation causes stress relaxation at the crack-tip region until a steady-state stress distribution is achieved. The steady-state stress is characterized by path-independent integral C*. Five different integral paths around the crack tip were considered and the average value of calculated C*-integrals was taken as final result [21].

**Figure 3:**
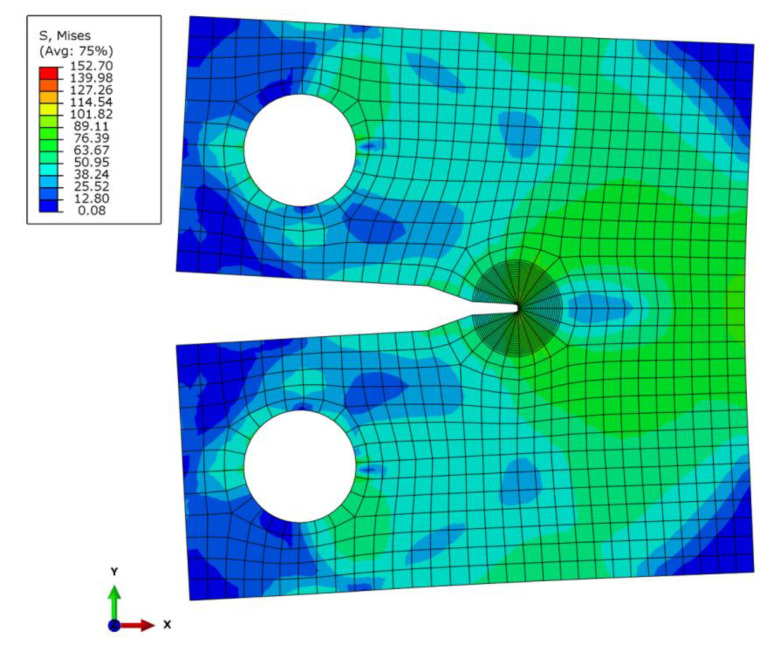
Distribution of von Mises stresses obtained by elastic-creep analysis.

To gain confidence in the present FE analysis, elastic and elastic-creep analyses of a homogeneous CT specimen were performed. Figure 3 shows the distribution of von Mises stresses obtained by elastic-creep analysis (deformation scale factor is 10). Table 2 compares the values of fracture mechanics factors *K*_I_ and C* obtained by FE analyses with analytical solutions for CT specimen [22,23]. It is evident that the FE results provide confidence in the FE analyses for elastic and steady-state creep conditions.

## 3. Results and Discussion

C* is estimated experimentally from measurements of creep load line displacement rate according from tests carried out on CT specimens based on the recommendations of ASTM E1457-15. The value of C* integral is determined using the following Equation [3]:

(4) $$C^{*}=\eta \frac{n}{n+1}\frac{F{\dot{V}}_{c}}{B\left(W-a\right)}\;,$$

where *F* denotes the applied constant load, *B* is the specimen thickness, ${\dot{V}}_{c}$. is the creep load line displacement rate, and *η* is an experimental calibration factor. The geometrical factor *η* depends only on the specimen geometry and the crack length *a*. For the CT specimen, the geometric factor *η* is determined according to the following expression:

(5) $$\eta =2+0.522(1-\frac{a}{W})$$

It is worth noting that Equation (4) is developed for homogeneous materials. This means that this equation is not applicable to heterogeneous materials such as welded joints. A directly measured ${\dot{V}}_{c}$ value from the welded CT specimen reflects a partial influence of material and geometry constraints. It is therefore necessary to extend the expression for estimating the C* integral (Equation (4)) that will take these constraints into account. It can be assumed that the modified equation for estimating the C* integral for a welded CT specimen has the following form:

(6) $$C^{*}=\eta \frac{n}{n+1}\frac{F{\dot{V}}_{c}}{B\left(W-a\right)}\varphi \;,$$

where *φ* denotes the calibration factor dependent on material and geometry constraints. It can be written as follows:

(7) $$\varphi =\varphi \left(MF_{\mathrm{WM}},\;MF_{\mathrm{HAZ}},W/h,a/W\right)\;,$$

This functional dependence will be found based on the results of extensive elastic-creep FE analyses performed. Factor *φ* values were calculated as the ratio of the C* integral of the heterogeneous (welded) CT specimen and the C* integral for the homogeneous CT specimen:

(8) $$\varphi =\frac{{\left(C^{*}\right)}_{HET}}{{\left(C^{*}\right)}_{HOM}}\;,$$

**Figure 4:**
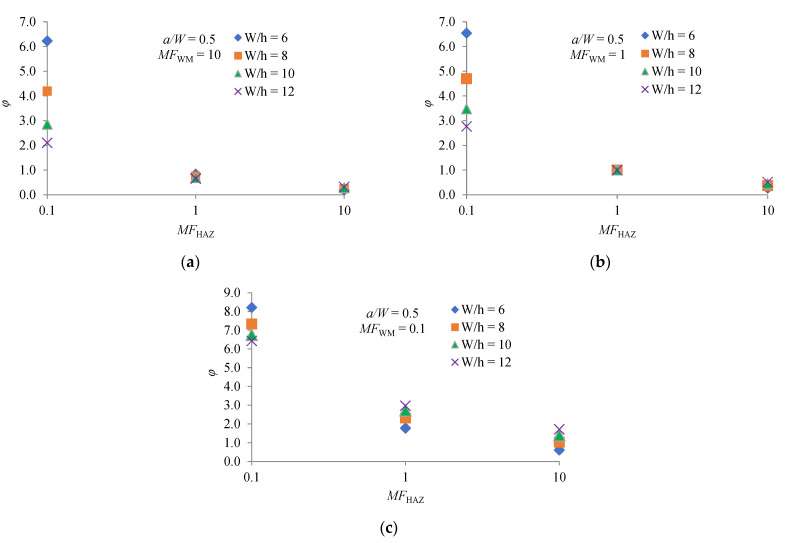
Dependence *φ* factor on *MF*_HAZ_, *MF*_WM_ and *W/h* for *a/W* = 0.5: (**a**) *MF*_WM_ = 10; (**b**) *MF*_WM_ = 1; (**c**) *MF*_WM_ = 0.1.

Figure 4a–c show the influence of *MF*_HAZ_, *MF*_WM_ and *W/h* on the factor *φ* for the ratio *a/W* = 0.5. It can be seen that *MF*_HAZ_ has a strong influence on the factor *φ* for the considered values of *MF*_WM_ and *W/h*. Values of *φ* are highest for creep-soft HAZ (*MF*_HAZ_ = 0.1). It can be seen that these values are significantly higher than one, meaning that the C* integral is lower than the C* integral for a homogeneous CT specimen. A larger C* integral causes a higher rate of CCG. For a given value of *MF*_HAZ_, the factor *φ* is higher if *MF*_WM_ is lower. For creep-hard HAZ (*MF*_HAZ_ = 10), the values of *φ* are mostly lower than one, meaning that the C* integral is less than the C* integral for a homogeneous CT specimen. The influence of *W/h* on the *φ* factor is significant for creep-soft HAZ. The factor *φ* is higher for the larger HAZ width *h* (lower *W/h* ratio).

**Figure 5:**
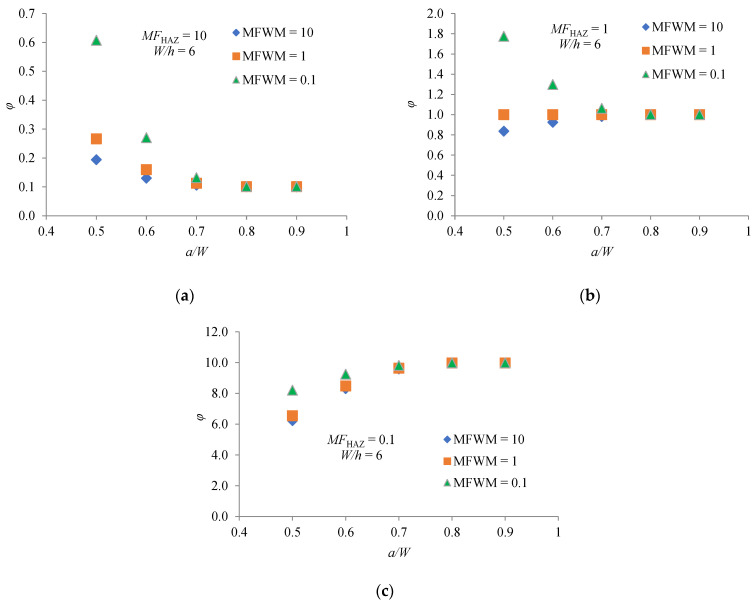
Dependence *φ* factor on *MF*_HAZ_, *MF*_WM_ and *a/W* for *W/h* = 6: (**a**) *MF*_HAZ_ = 10; (**b**) *MF*_HAZ_ = 1; (**c**) *MF*_HAZ_ = 0.1.

A similar analysis can be performed for the other *a/W* values considered. However, for easier analysis, Figure 5a–c show the dependence *φ* factor on the *a/W* ratio for the considered values of *MF*_HAZ_ and *MF*_WM_ at the ratio *W/h* = 6.

It can be seen that effects of *MF*_HAZ_ on *φ* are quite complex. For creep-hard HAZ (*MF*_HAZ_ = 10), the values of *φ* are lower than one for all considered values of *a/W*. The value of *φ* becomes lower and asymptotically approaches a constant value as *a/W* increase. The value of *MF*_WM_ has almost no effect on *φ* when *a/W* ≥ 0.7.

For creep match HAZ (*MF*_HAZ_ = 1), the values of *φ* in the range 0.5 ≤ *a/W* ≤ 0.7 are higher than one if WM is creep-soft material (*MF*_WM_ = 0.1). On the other side, for creep-hard WM (*MF*_WM_ = 10) and range 0.5 ≤ *a/W* ≤ 0.7 the values of *φ* are lower than one. In both cases, for values of *a/W* > 0.7, the value of *φ* asymptotically approaches one. For creep match WM the value of *φ* is one for all considered values of *a/W*. This is expected because it is in fact a homogeneous material.

For creep-soft HAZ (*MF*_HAZ_ = 0.1), the values of *φ* are significantly higher than one for all considered values of *a/W*. The value of *φ* becomes higher and asymptotically approaches a constant value as *a/W* increase. The value of *MF*_WM_ has almost no effect on *φ* when *a/W* ≥ 0.7.

**Figure 6:**
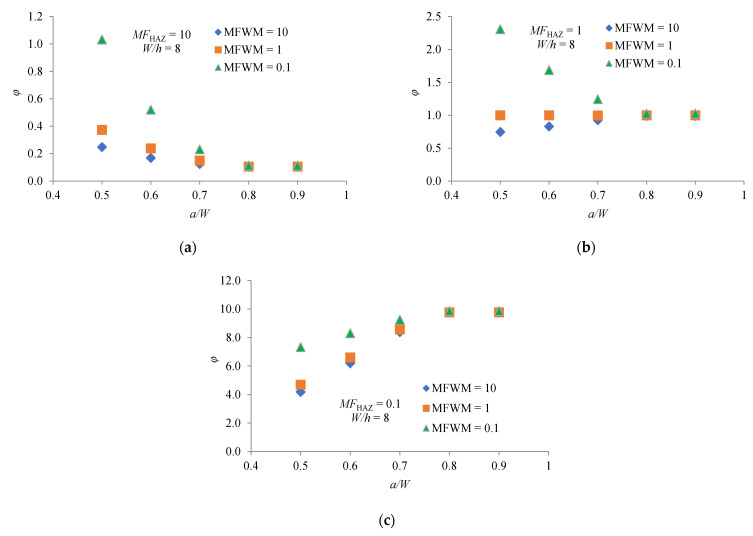
Dependence *φ* factor on *MF*_HAZ_, *MF*_WM_ and *a/W* for *W/h* = 8: (**a**) *MF*_HAZ_ = 10; (**b**) *MF*_HAZ_ = 1; (**c**) *MF*_HAZ_ = 0.1.

A similar analysis can be performed for the other *W/h* values considered. Figure 6a–c show the dependence *φ* factor on the *a/W* ratio for the considered values of *MF*_HAZ_ and *MF*_WM_ at the ratio *W/h* = 8. Thus, for *a/W* = 0.5 and *MF*_HAZ_ = 10, the corresponding values of *φ* for *W/h* = 8 are higher than those for *W/h* = 6. Likewise, for *a/W* = 0.5 and *MF*_HAZ_ = 0.1, the corresponding values of *φ* for *W/h* = 8 are lower than those for *W/h* = 6. In both cases, the value of *φ* asymptotically approaches a constant value as *a/W* increase. For *a/W* = 0.5 and *MF*_HAZ_ = 1 and if WM is creep-soft material (*MF*_WM_ = 0.1), the value of *φ* for *W/h* = 8 is higher than this for *W/h* = 6. For *a/W* = 0.5 and *MF*_HAZ_ = 1 and if WM is creep-hard material (*MF*_WM_ = 10), the value of *φ* for *W/h* = 8 is lower than this for *W/h* = 6. In the case where WM and HAZ are creep match materials, the value of *φ* is one for all considered *a/W* ratios.

Considering the dependence of the factor *φ* on *MF*_HAZ_, *MF*_WM_ and *a/W* for all *W/h* ratios, it can be generally concluded that there is a strong and complex influence of material and geometry constraints on the CCG rate. If the HAZ creep is a soft material, which is often the practice [18], the factor *φ* will have values higher than one. This means that the C* integral will be higher than the C* integral for homogenic material, so the CCG rate will also be higher. It is clear that a higher CCG rate means a shorter lifetime of the welded structure.

There is a unique relationship between the C* integral and the crack-tip stress and strain rate fields as follows [24]:

(9) $$\sigma_{ij}={\left(\frac{C^{*}}{I_{n}Ar}\right)}^{\frac{1}{1+n}}{\hat{\sigma }}_{ij}\left(\theta ,n\right)\;,$$

(10) $${\dot{\varepsilon }}_{ij}={\left(\frac{C^{*}}{I_{n}Ar}\right)}^{\frac{n}{1+n}}{\hat{\varepsilon }}_{ij}\left(\theta ,n\right)\;,$$

where *r* is distance from the crack tip, *I_n_* is quantity that depends on exponent *n* and the stress condition, ${\hat{\sigma }}_{ij}$. and ${\hat{\varepsilon }}_{ij}$ are angular functions. Analyzing the diagrams in Figure 4, Figure 5 and Figure 6, it can be clearly concluded that the values of *φ* change with different combination of *MF*_HAZ_, *MF*_WM_, *W/h* and *a/W*. Different combinations of these parameters change the crack-tip constraint effect, and thus the magnitude of the crack-tip stress and strain rate. A higher magnitude of crack-tip stress and strain rate means a higher value of the C* integral, and thus a higher value of *φ*.

Using the results of the analyses performed and applying the software package TuringBot [25], an equation was found which can satisfactorily estimate *φ* values for the considered values of *MF*_HAZ_, *MF*_WM_, *a/W* and *W/h*. This is the following expression:

(11) $$\varphi =\frac{a/W}{MF_{\mathrm{HAZ}}}+\frac{2}{MF_{\mathrm{HAZ}}+MF_{\mathrm{WM}}\left(W/h\right)}\;\;,$$

The *R*-squared for this model is 0.96 and the RMS error is 0.74. This confirms the high goodness-of-fit of the selected model.

## 4. Conclusions

Based on systematic FE analyses, a modified equation was proposed to estimate the C* integral for the welded CT specimen with a crack located in the center of the HAZ. Compared to a homogeneous CT specimen, creep-soft HAZ gives higher values of the C* integral while creep-hard HAZ reduces the value of the C* integral. This means that the existing equation for the C* integral in ASTM E 1457 may underestimate or overestimate the actual value of the C* integral for the welded CT sample. The influence of *W/h* on the C* integral is significant for creep-soft HAZ. The C* integral is higher for the larger HAZ width *h* (lower *W/h* ratio). Finally, an expression for estimating the C* integral for the mismatched welded CT specimen is proposed.

## Figures and Tables

**Table 1:** Considered material mismatches.

*MF*_WM_	*MF*_HAZ_
10	0.1
10	1
10	10
1	0.1
1	1
1	10
0.1	0.1
0.1	1
0.1	10

**Table 2:** Ratios of the FE *K*_I_ and C* with analytical solutions.

*K*_I_(FE)/*K*_I_		1.005
C*(FE)/C*	*n* = 5	0.978
	*n* = 10	0.982
